# Domestic burns that occurred during the COVID-19 pandemic in Brazil: a descriptive cross-sectional study

**DOI:** 10.1590/1516-3180.2021.0888.R1.22022022

**Published:** 2022-09-12

**Authors:** Raquel Pan, Paola Maria Freitas dos Santos, Isabella Luiz Resende, Kleiton Gonçalves do Nascimento, José Adorno, Marco Túlio Rodrigues da Cunha, Noélle de Oliveira Freitas

**Affiliations:** I PhD. Adjunct Professor, Department of Nursing in Hospital Assistance, Universidade Federal do Triângulo Mineiro (UFTM), Uberaba (MG), Brazil.; II RN. Resident, Program of Multiprofessional Integrated Residency in Health of Elderly, Universidade Federal do Triângulo Mineiro (UFTM), Uberaba (MG), Brazil.; III RN. Master Student, Health Care Graduate Program, Universidade Federal do Triângulo Mineiro (UFTM), Uberaba (MG), Brazil.; IV PhD. Administrative Nurse Technician, Department of Nursing in Hospital Assistance, Universidade Federal do Triângulo Mineiro (UFTM), Uberaba (MG), Brazil.; V MSc. Coordinator, Burns and Plastic Surgery Service, Santa Lucia Hospital, Brasilia (DF), Brazil.; VI PhD. Adjunct Professor, Department of Clinical Surgery, Universidade Federal do Triângulo Mineiro (UFTM), Uberaba (MG), Brazil.; VII PhD. Professor, Postgraduate Program in Nursing, Universidade de Guarulhos (UNG), Guarulhos (SP), Brazil.

**Keywords:** Burns, Accidents, home, Accident prevention, Pandemics, COVID-19, Burn injury, Health care, Lockdown, Social distancing, Survey study

## Abstract

**BACKGROUND:**

Due to “stay at home” restrictions during the coronavirus disease 2019 (COVID-19) pandemic, people spent more time at home leading to an increase in home accidents, including burns.

**OBJECTIVE:**

To investigate the epidemiology of burns that occurred within homes during the COVID-19 pandemic in Brazil.

**DESIGN AND SETTINGS:**

This was a quantitative, descriptive, and cross-sectional study with a non-probabilistic sample.

**METHODS:**

Data were collected through the distribution of survey links on social networking sites and websites, and through email between December 2020 and February 2021. Participants were over 18 years of age, living in Brazil. Data analysis was performed using descriptive and dispersion statistics.

**RESULTS:**

A total of 939 adults (aged > 18 years) participated in this study. The mean age was 37.2 years (standard deviation [SD] = 12.5), 75.6% were female, 70.0% self-reported white skin color, 74% had completed higher education, and 28.1% had an income of 3 to 6 times the monthly minimum wage. A total of 21.6% suffered burns during the pandemic, 44.3% from a hot object. Approximately 49.3% never had access to a burn prevention campaign.

**CONCLUSION:**

It is necessary to develop burn prevention strategies that reach a wider population and to strengthen public policies to reduce the prevalence of domestic burns, especially during the pandemic.

## INTRODUCTION

Burns injuries are a universal health hazard, which carry high morbidity and mortality, especially in low- and middle-income countries.^1^ The etiology, time of exposure to the thermal agent, and depth of the burn wounds influence the severity of the injury.^2^ In addition to physical injuries, burns have psychological, economic, and social implications of great impact and are considered a global public health problem. Among the causes of burns, the most frequent are exposure to flames, scalding, and electrical and chemical burns.^3^

It is estimated that burns are responsible for about 180,000 deaths annually, with the highest incidence in low- and middle-income countries, with approximately two-thirds occurring on the African and Asian continents.^1^ In the United States of America, there were more than 410,000 cases of burns in 2008 and around 40,000 incidents required care at the hospital level.^1^ In Brazil, the annual estimates of burn injuries can be as high as 1,000,000 incidents.^4^ Several factors influence the occurrence of burns, such as socioeconomic level, geographic distribution, age, and gender.^5^

To reduce the burden on healthcare during the coronavirus disease 2019 (COVID-19) pandemic, “stay at home” orders and “lockdowns” were implemented at different stages worldwide with varying lengths of time and severity. This raised the concern that domestic accidents, mainly burns, might increase during this period.^6^ The need to adopt social distancing and lockdowns affected family routines and had a negative impact on individuals from a social and psychological point of view.^7^

To adhere to the World Health Organization (WHO) measures related to COVID-19, in March 2020, the over-the-counter sale of hand sanitizers with 70% alcohol in liquid or gel form was approved in Brazil.^8^ This public health measure was necessary to prevent the spread of disease in cases where it would be impossible to sanitize hands with soap and water. However, its sale and use were not accompanied by educational campaigns on safety for the use of products with such high alcohol content. The improper use of alcohol-based sanitizers has put families at risk for burns and caused an increase in the number of hospitalizations.^9^ Between March 19 and August 12, 2020, there were 497 hospital admissions related to hand sanitizers with 70% alcohol, in 36 Burn Treatment Centers (BTC) and general hospitals across the country.^10^ The concerns were related to the inappropriate use of alcohol in liquid and gel form for sanitizing purposes, and the various situations that triggered accidental burn injuries at home, such as contact with hot liquids, fire, and hot objects.

The Brazilian Society of Burns reported that before the pandemic, a majority of burn accidents already occurred in homes (70%), with children comprising 40% of cases.^11^ The lockdowns and social distancing measures affected the family environment and their habits. For example, people had to cook more frequently at home, and in families with children, parents had to work from home while supervising their children during the day. Hence, it should be expected to find an increase in preventable burn injuries taking place at home as a result of the COVID-19 pandemic restrictions in Brazil.

## OBJECTIVE

The aim of this study was to investigate burns that occurred within homes during the COVID-19 pandemic in Brazil.

## METHODS

### Study design

This study adopts a quantitative, descriptive, and cross-sectional design with a non-probabilistic sampling method that aims to survey patterns of burn injuries occurring at home throughout Brazil. Data were collected using the questionnaire-based survey method.^12^

This paper was written in accordance with the guidelines for reporting observational epidemiological studies (STrengthening the Reporting of OBservational studies in Epidemiology, STROBE).

### Procedure for data collection

In the first phase of the study, a 25-item questionnaire (entitled Investigation of burn injuries that occurred during the COVID-19 pandemic) was designed. The instrument was divided into two sections: *General sociodemographic items*, including 11 questions about sex, date of birth, skin color, education, profession, city and state of residence, number of people living in the same house with the participant, number of people under 18 years of age living with the participant, email and salary per month (R$ 1.100 was the Brazilian minimum wage in 2021.^13^ As a point of comparison, the US dollar was valued at 5.19 Real in August 30, 2021^14^); and *Specific questions regarding domestic burns that occurred during the COVID-19 pandemic*, the first two questions were about the occurrence of domestic burns – if the participant had not suffered burns, he/she was sent to the last question about burn prevention campaigns – the following 11 items were about the location and date of occurrence, if there were multiple episodes of burns, cause of the burn, social distancing, first aid, hospitalization, length of stay, skin graft, burn prevention campaign.

In the second phase, an expert committee was constituted of four healthcare professionals (two physical therapists, one doctor, and one nurse; a fifth was invited but did not respond) with expertise in the area of burn injuries, to validate the instrument. An email invitation was sent to each member of the expert committee with the study objectives, study protocol, their role, and the link to evaluate the instrument built using the SurveyMonkey software (San Mateo, California, United States). The committee assessed the questions for content and apparent validation. The evaluation was based on the theory of elaboration of psychological scales.^15^ The total instrument Content Validity Index (CVI) value was 0.98 and only one item had a value of 0.75 (“If you were hospitalized more than once due to burns, please specify”). In some questions, changes suggested by the experts were incorporated, such as living in urban or rural areas, type of housing, parts of the body that were burnt, difference between barbecue area and leisure area, treatment performed at home, and in the health service. The authors reviewed the new version and included marital status as an item to be analyzed. Thus, a new version of the instrument (33 items) was developed and submitted for reevaluation to three members of the expert committee. The modified version of the instrument had a CVI value of 0.99 and all items, a value above 0.80. No further changes were suggested and the burn investigation instrument was considered validated by the committee of experts.

In the third phase, the survey link was sent through social networking sites, websites, and email, with the aim of reaching the largest possible number of participants in the national territory. A database was created containing the emails of federal and private institutions, including hospitals, found using Google search tools. A designated email account for the research study was created, and invitations to participate were sent from this address. To expand the sample size, the first guests to participate in the survey were invited to forward the link to their personal contacts, generating a snowball sample. The survey was available online for the period of December 2, 2020, to February 15, 2021.

### Inclusion and exclusion criteria

The study selected participants who were aged 18 years and older, residing in Brazil during the period under study, spoke Portuguese, had Internet access, and were Internet literate.

Instruments answered by participants under the age of 18, duplicates, or responses with a coherence error that invalidated the instrument were excluded from the study.

### Data analysis

For the analysis of the apparent and content validation data of the instrument, the CVI was calculated.^16,17^ To calculate the CVI, a Likert-type scale with scores from one to four was used, which assessed the relevance/representativeness of the responses identified as follows: 1 = not relevant or not representative (strongly disagree), 2 = item needs major review to be representative (disagree), 3 = item needs minor revision to be representative (agree), and 4 = relevant or representative item (strongly agree). The answer “I don’t know” was assigned a value of 0. The following formula was used: the number of responses with scores of 3 or 4 divided by the total number of responses in the script. Items that received a score of “1” or “2” were revised and responses marked as “I don’t know” were eliminated.^16,17^ To calculate the instrument’s total CVI, the average of the values of the items calculated separately was considered, that is, the sum of all the CVI calculated separately and the division by the number of items considered in the evaluation.^18^ A CVI value above 0.80 was considered the minimum.^19,20^

Data collected in the third phase of the study through SurveyMonkey were exported to IBM SPSS Statistics for Windows, version 23 (IBM Corp., Armonk, New York, United States). Descriptive and dispersion analyses were performed for all variables.

### Ethical considerations

The research protocol was registered and approved by the Human Research Ethics Committee on November 1, 2021^21^ (number 34396320.4.0000.5154).

The participants had access to the Informed Consent Form (ICF) on the first page of the instrument and could only view the questionnaire after agreeing to the ICF conditions. Electronic signatures on the ICF were collected from all individuals involved in the study.

## RESULTS

Over the study period (2 and a half months), 939 participants completed the survey. The sociodemographic profile showed that 710 were female (75.6%), and there was a predominance of respondents who declared themselves as having white skin color (70.0%). The mean age of the participants was 37.2 years (standard deviation, SD = 12.5). Regarding the level of education among the participants, 74% declared that they had completed higher education, and 28.1% had an income of 3 to 6 times the minimum wage. A total of 42.6% were married and 97.2% lived in an urban area (Table 1).

**Table 1.** Distribution of sociodemographic variables of participants in the study on burns in the pandemic carried out in Brazil (n = 939)

Variables	Categories	n	%
Gender	Female	710	75.6
	Male	225	24.0
	Other	4	0.40
Skin color	White	657	70.0
	Brown	207	22.0
	Black	55	5.8
	Yellow	18	1.9
	Indigenous	2	0.2
Age, mean (SD)^a^		939	37.2 (12.5)
Education	Complete primary education	7	0.7
	Incomplete elementary school	5	0.5
	Complete high school	58	6.2
	Incomplete high school	6	0.6
	Complete higher education	695	74.0
	Incomplete higher education	168	17.9
Marital status	Married	400	42.6
	Divorced/Separated	70	7.5
	Single	387	41.2
	Stable union	73	7.8
	Widower/Widow	9	1.0
Income	Less than 1 minimum wage^b^	113	12.0
	From 1 to 3 times the minimum wage	261	27.8
	From 3 to 6 times the minimum wage	264	28.1
	From 6 to 9 times the minimum wage	129	13.7
	From 9 to 12 times the minimum wage	82	8.7
	Greater than 13 times the minimum wage	90	9.6
Residence	Urban area	913	97.2
	Countryside	21	2.2
	Other	5	0.5

SD = standard deviation. ^a^standard deviation; ^b^R$ 1.100 – value of the Brazilian minimum wage paid monthly in 2021 (1 United States Dollar = 5,19 Real). SD = standard deviation.

Two hundred and one participants (21.6%) sustained burn injuries, of whom 173 (76.1%) were female and 156 (77.6%) identified themselves as having brown skin color. In terms of occupation, the majority comprised 93 (46.3%) teachers, and 65 (32.3%) persons engaged in public service. The mean age was 37.2 years (SD = 12.87), 159 (79.1%) had an incomplete higher education level, 144 (71.6%) were single, 161 had an income of at least 9 times the minimum wage or higher, and all lived in urban areas (Table 2).

**Table 2.** Distribution of sociodemographic variables of study participants who suffered burns in the pandemic in Brazil (n = 201)

Variables	Categories	n	%
Gender	Female	153	76.1
	Male	46	22.9
	Other	2	1
Skin color	Brown	156	77.6
	Black	45	22.4
Age, mean (SD)^a^			37.22 (12.87)
Education	Complete higher education	42	20.9
	Incomplete higher education	159	79.1
Marital status	Single	144	71.6
	Stable union	57	28.4
Income	From 6 to 9 times minimum wage^b^	40	19.9
	From 9 to 12 times minimum wage^b^	81	40.3
	Greater than 13 times minimum wage^b^	80	39.8
Residence	Urban area	201	100
Occupation	Teacher	93	46.3
	Psychologist	13	6.5
	Chemical	3	1.5
	Secretary	8	4.0
	Public server	65	32.3
	Sociologist	2	1.0
	Nursing technician	17	8.5

^a^standard deviation; ^b^R$ 1.100 - value of the Brazilian minimum wage paid monthly in 2021 (1 United States Dollar = 5,19 Real).

The survey identified that other family members had also sustained burn injuries (117 responses, 58.2%) during the pandemic period (Table 3). The following body areas were identified as being involved: right hand, 82 (40.2%); left hand, 42 (20.6%); and right arm, 33 (16.1%). The agents causing the burns were most frequently hot objects (44.3%) and hot liquids (29.9%) and the kitchen was the area of the house where most injuries occurred (77.1%).

**Table 3.** Distribution of clinical and environmental characteristics of study participants who suffered burns during the pandemic in Brazil (n = 201)

Variables	Categories	n	%
Family member burns	No	84	41.8
	Yes	117	58.2
Part of the body affected	Right hand	82	40.2
	Left hand	42	20.6
	Right arm	33	16.1
	Left arm	18	8.8
	Left leg	11	5.4
	Face	10	4.9
	Abdomen	8	3.9
	Right foot	5	2.5
	Right leg	4	1.9
	Neck	4	1.9
	Thorax	4	1.9
	Scalp	1	0.5
	Back	2	1.0
	Left foot	2	1.0
	Genital organs	0	0
Agent causing the burn	Hot object	89	44.3
	Hot liquid	60	29.9
	Vapor	11	5.5
	Direct flame	10	5.0
	Liquid alcohol	1	0.5
	Chemicals	1	0.5
	Did not answer	29	14.4
Location in the house where the burn occurred	Kitchen	155	77.1
	Bedroom	5	2.5
	Bathroom	1	0.5
	Barbecue grill	3	1.5
	Recreation Area	1	0.5
	Did not answer	36	17.9

The month with the highest incidence of burn-related accidents was November, followed by December and May 2020. Among the participants, 23 (11.4%) had more than one burn during the pandemic period, with a minimum interval of 7 days and maximum interval of 10 months between injuries.

Regarding the management of burn injuries, 168 participants (83.6%) did not seek care after the accident, and 171 (85.1%) did not require hospitalization (Table 4). The use of two products in the treatment of burns stands out: medicinal plants (9, 4.4%) and running water (9, 4.4%).

**Table 4.** Management of participants after the occurrence of burns during the pandemic in Brazil (n = 201)

Variables	Categories	n	%
Seek medical attention	No	168	83.6
	Yes	4	2.0
	Did not answer	29	14.4
Hospital internment	No	171	85.1
	Yes	1	0.5
	Did not answer	29	14.4
Specific product for burns in the healthcare unit	Yes	47	23.4
	No	65	32.3
	Did not answer	89	44.3
Used some product at home in the burn	Yes	86	42.8
	No	24	11.9
	Did not answer	91	45.3
Products used on burns at home	Medicinal plant	9	4.4
	Running water	9	4.4
	Oil	3	1.5
	Toothpaste	3	1.5
	Saline	2	1.0
	Ice bag	2	1.0
	Egg white	1	0.5
	Ozonized oil	1	0.5
	Laser therapy	1	0.5

Of the participants who had sustained a burn injury, 99 (49.3%) reported that they were unaware of any burn prevention campaigns. Regarding the means of accessing information on burn prevention, 19 (26.4%) reported being healthcare professionals themselves and thus knowing how to prevent burn accidents, while articles in the media (TV programs and radio) represented the other substantial source of awareness (Table 5).

**Table 5.** Profile of preventive measures adopted by participants on burn prevention in Brazil (n = 201)

Variables	Categories	n	%
Have you seen any burn prevention campaigns?	No	99	49.3
	Yes, before the pandemic	64	31.8
	Did not answer	38	18.9
Means of communication used to prevent burns	You are a health professional	19	26.4
	Articles in media such as TV or radio programs	18	25.0
	Guidelines from health professionals	10	13.9
	Other	8	11.1
	Scientific article readings	6	8.3
	You have already suffered burns in the past and from this, you follow strategies to prevent further burns	6	8.3
	Someone you know suffered burns and from there you follow burn prevention strategies	3	4.2
	Profiles on social networks such as Facebook, Instagram, and Twitter	2	2.3

In relation to other means used to obtain information about burns, the word “prevention” appeared 16 times in phrases such as the following: normal preventive measures linked to attention and care, equipment installed by trained professionals; prevention strategies given by older family members, and previous knowledge about preventive measures.

Regarding measures of social distancing during the pandemic, 52 (25.9%) participants remained in total isolation for the entire period, in contact exclusively with people who stayed in the same house as them, while 33 (16.4%) remained in complete isolation for the first three months of the pandemic; 13 participants (6.5%) remained in partial isolation for the first three months of the pandemic, meeting a few people other than those who lived in the same house as them; 58 (28.9%) remained in partial isolation throughout the pandemic; 8 (4.0%) did not practice social distancing at any time; 3 (1.5%) did not want to answer, and 34 (16.4%) did not answer.

## DISCUSSION

Due to the measures imposed during the COVID-19 pandemic, the risks of the population experiencing traumatic burn injuries while in isolation are considerably higher.^22^ In Brazil, burn injuries resulted in around 26,173 hospital admissions in 2020, a majority being in the Southeast area with 9,298 cases and 745 deaths.^23^ A previous study evaluating the number of hospitalizations and deaths caused by burn injuries in pre-pandemic Brazil, identified the South and Midwest regions as having higher hospital admission rates.^24^ During the COVID-19 pandemic in 2020, Brazil had a high number of deaths due to burns when compared to other countries.^24^

According to data from the Brazilian Institute of Statistics and Geography,^25^ women constitute 51.03% of the country’s population and 84.4% of the total population live in urban areas. The representation of ethnic-racial background identified in this study is similar to the frequency in the Brazilian population, in which white skin color accounts for 47.7% and brown, 43.1%.^26^

The average age of the participants who sustained burn injuries was 37.22 years. In a study comparing burn cases between March 16 and September 30, 2019, with the same period in 2020, it was shown that the average age of patients who suffered burns increased from 38.2, before the pandemic, to 55.7 in 2020. This indicates that adults are the most affected by burns, even during the pandemic.^22^

In this study, 40.3% of the participants who suffered from burns during the pandemic were of a high socioeconomic status. This differs from another survey conducted over a 10-year period at the Burn Care Center of the Institute of Medical Sciences in Pakistan, where a total of 94,664 people were assessed and the majority of patients who suffered burn injuries were of low socioeconomic status.^27^ It is believed that low-income people are more vulnerable to burns because of the lack of guidance on care and prevention of burns and low access to sources of media. Thus, this study suggests a need for reflection on the prevention strategies against burns and the means of dissemination, as even though the participants in this research belonged to the highest socioeconomic status, this did not prevent them from having burn accidents.

The hands were the most affected body part, followed by the arms. In a previous study, it was highlighted that the upper limbs and trunk were the most affected in the profile of burn victims.^28^ Another study conducted an analysis of medical records of patients in the Burns Care Unit in the Northeast of the country from June 2015 to July 2016, in which of the 86 records reported the affected body area, the regions of the upper limbs were the most affected (51.2%) as an area of greater exposure during household chores.^29^

The most prevalent causal agents were burns caused by hot objects (44.3%) and liquids (29.9%). A study conducted with data from Uruguay^30^ found direct fire (71%), followed by hot liquids (9%) and electricity (5%) to be the primary causes of burn injuries, and another study conducted at a regional adult burn center in the United Kingdom from March 23 to May 6, 2020, determined a prevalence of flame and scalding in burn accidents.^31^ In a survey conducted in an emergency care hospital in the Northeast region of Brazil, the incidence of burns caused by hot liquids increased from January to September 2020 compared to the previous year, with other causes being flames and chemical substances.^32^ These findings differ from the present study, as factors such as social distancing to reduce COVID-19 transmission caused a change in family routines, evidenced by the change in the profile of burns in Brazil.

The present study showed that burns occurred more often in kitchens. Other international studies involving 3,050 patients also identified that burns in domestic environments accounted for 57% of cases attended,^30^ and 71.3% of all accidents causing burns.^27^

Prevention campaigns that increase population awareness about the risks and behaviors leading to burns are the best strategy to reduce such injuries, hospital admissions, and deaths. A study involving 776 people from the Southern region of Brazil demonstrated that, with respect to prevention, only 31.44% of patients claimed to have received information from public campaigns.^33^ Similarly, our study identified that 99 (49.3%) participants had not seen information from public burn prevention campaigns and 64 (31.5%) had access to them before the pandemic. The findings from both studies suggest that there are opportunities to improve the dissemination of public burn prevention campaigns in Brazil.

This study had some limitations. First, the online survey distribution and data collection did not reach all population groups. Data from the Brazilian Institute of Geography and Statistics (Instituto Brasileiro de Geografia e Estatística, IBGE) in 2019 show that 82.7% of Brazilian households have internet access in urban areas while the rural distribution is only 55.6%.^34^ Another survey conducted by the Regional Center for Studies on the Development of the Information Society (Cetic.br) published in 2020, identified that 99% of households of higher socioeconomic status have access to the internet, while 50% of households of lower socioeconomic status have this facility.^35^ Based on existing evidence in the literature,^35^ it was expected that the profile of people who suffered burn accidents during the pandemic would be from low-income settings. However, the profile of people in the current research project was different because the methods used to disseminate the survey were more likely to reach people of higher socioeconomic status. People who live in low-income settings may not have access to the Internet or lack technical knowledge about its use, and thus, may not have had the opportunity to participate in this survey. Nonetheless, the study highlighted that people of higher socioeconomic status also suffered from burn injuries. Second, the email sent may have been marked as spam mail and may not have been seen by the respondent. Assuming that keeping the questionnaire available for a longer period of time increases the likelihood that people who occasionally access the Internet will participate in the survey, this study may have compromised on a higher number of participants from regions of Brazil other than the southeast, since data collection took place over a short period of time, given the urgency of the theme.

## CONCLUSION

This study identified that during the COVID-19 pandemic in Brazil, a majority of the people who sustained burn injuries were female, had brown skin color, and had incomplete higher education. Almost half of the participants suffered burns from hot objects or hot liquids, and most accidents occurred in the kitchen. Almost half of the participants reported that they had never accessed a burn prevention campaign. Thus, this study proposes improving the methods of disseminating educational campaigns and guidance on burn prevention, in addition to supporting public health policies to guide the population in an attempt to reduce the incidence of domestic burns. Future studies should identify risk factors associated with burns at home to inform future burn prevention initiatives.
